# Peri-Operative Anesthetic Management of Left Atrial-Esophageal Fistula

**DOI:** 10.7759/cureus.65520

**Published:** 2024-07-27

**Authors:** Nasim Shakibai, Sheryar Muhammad

**Affiliations:** 1 Anesthesiology, Oakland University William Beaumont School of Medicine, Royal Oak, USA; 2 Anesthesiology, Corewell Health William Beaumont University Hospital, Royal Oak, USA

**Keywords:** atrial fib ablation, oxygenation, one lung ventilation, cpap utilization, atrial-esophageal fistula

## Abstract

Atrial-esophageal fistulas are rare and potentially fatal complications that can occur from radiofrequency ablation for the treatment of atrial fibrillation. Due to the proximity of the right atrium to the esophagus, thermal injuries can involuntarily lead to connections between the heart and esophagus. In this case study, a 67-year-old male developed an atrial-esophageal fistula following atrial fibrillation ablation. After discharge, the patient first presented with melena with a range of complications including aspiration, fever, atrial fibrillation, and neurological symptoms. The fistula was repaired promptly after diagnosis requiring meticulous planning by the anesthesia and surgical teams. The major consideration from anesthesiology was providing adequate oxygenation during one-lung ventilation via continuous positive airway pressure on the non-dependent lung. This case also highlights the need for recognizing and managing potential complications associated with catheter ablation procedures. A thorough understanding of these rare but critical events is essential for optimizing patient outcomes and minimizing mortality rates, and physicians and healthcare professionals must remain vigilant regarding such complications.

## Introduction

Atrial-esophageal fistulas are a rare complication arising from atrial fibrillation radiofrequency ablation. They have an incidence of about 0.1%. This intervention can lead to thermal injuries affecting adjacent structures such as the esophagus as it is proximal to the site of endocardial ablation. Such injuries carry the risk of causing cerebral air embolism, gastrointestinal bleeding, and septic shock, often resulting in mortality rates ranging from 40% to 100% [1-2]. Current therapeutic modalities primarily involve surgical repair and placement of esophageal stents. 

This case report discusses a patient diagnosed with an atrial-esophageal fistula following radiofrequency ablation. Early detection and prompt treatment initiation enhanced the patient's chances of survival. We will discuss the signs and symptoms, treatment options, and anesthesia considerations related to atrial-esophageal fistulas. This case emphasizes the importance of meticulous preoperative planning, discusses different approaches to handling hypoxia during one-lung ventilation, and clarifies the risks of radiofrequency catheter ablations. 

## Case presentation

A 67-year-old male with a pertinent medical history of obstructive sleep apnea, a prior history of tobacco use, a remote esophageal ulcer, and atrial fibrillation managed with metoprolol and rivaroxaban, underwent atrial fibrillation ablation. The procedure was performed under general anesthesia, utilizing endotracheal intubation and esophageal temperature monitoring. The peak temperature recorded during the surgery was 35.78 degrees Celsius. Following the procedure, he experienced no complications and was discharged home on the same day. 

He presented to the emergency department (ED) seven days later with complaints of melena. Notably, he had taken his rivaroxaban dose the night before. There was a decrease in his hemoglobin level, dropping from a baseline of 13 to 10.8. Consequently, he received a transfusion of two units of packed red blood cells in the ED, and four-factor complexes were administered for the reversal of rivaroxaban. Subsequently, he underwent an esophagogastroduodenoscopy (EGD). During the procedure, intubation was challenging due to bleeding from the esophagus obscuring the airway, raising concerns for aspiration. Further examination revealed bleeding from an ulcer in the lower third of the esophagus, necessitating the placement of four stents. 

Following the procedure, he remained intubated due to the possibility of aspiration during intubation, prompting a bronchoscopy which confirmed the presence of aspirated blood and clots. Post procedure, he developed a fever and was initiated on piperacillin-tazobactam and vancomycin. Additionally, he experienced episodes of atrial fibrillation with rapid ventricular response, for which labetalol was initiated. Due to a subsequent drop in blood pressure, he was transitioned to an amiodarone drip and briefly required pressors. Over the course of several days, his condition improved, he was extubated and later discharged. 

**Figure 1:**
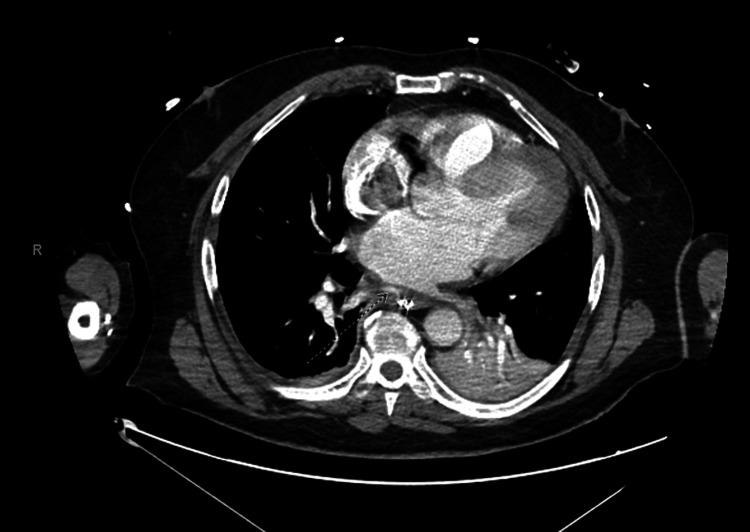
Computed tomography angiography of chest/abdomen/pelvis with contrast showing the atrial esophageal fistula

**Figure 2:**
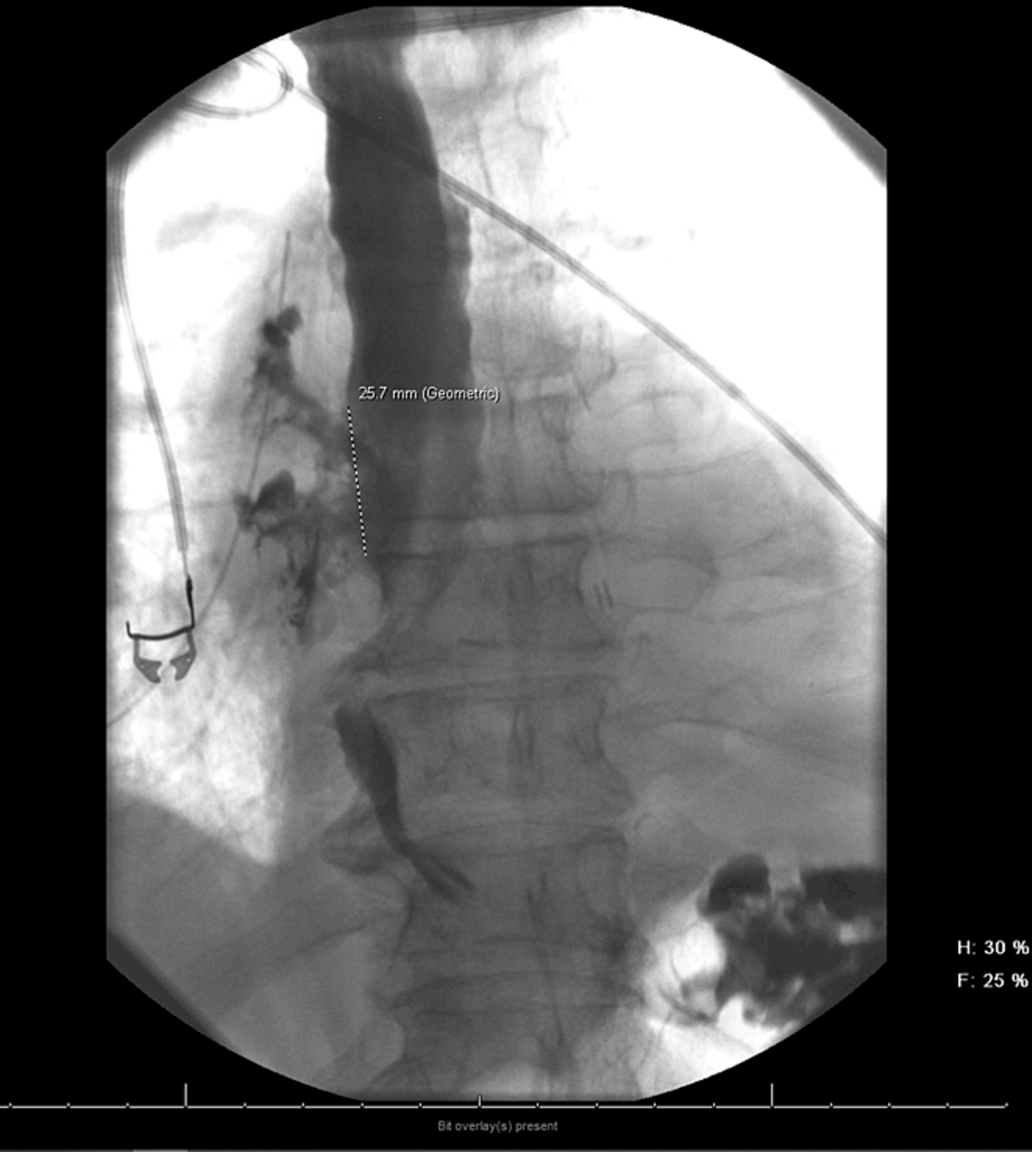
Esophageal barium swallow showing irregularity in the mid posterior wall of the esophagus, associated with a large leak if contrast in the mediastinum

Five days post discharge, he presented with acute left-sided weakness, near syncope, and palpitations, which spontaneously resolved. Upon evaluation, atrial fibrillation with rapid ventricular response was diagnosed, and he was initiated on a diltiazem drip. A computed tomography (CT) scan of the head ruled out a stroke. Shortly after, he became unresponsive and required intubation. Subsequent CT and barium swallow (Figures 1, 2) revealed an atrial esophageal fistula, implicating an air embolus as the likely cause of his transient neurological symptoms. A right thoracotomy was performed to repair the atrial-esophageal fistula, with significant emphasis placed on preoperative planning. Detailed discussions among the surgical and anesthesiology teams ensured alignment regarding the procedure's execution and anticipated outcomes, with the perfusionist team on standby. Anticipating the possibility of cardiopulmonary bypass during the procedure, femoral venous and arterial access were established preemptively prior to surgery, in readiness for a potential cannulation. Furthermore, his endotracheal tube was exchanged for a double-lumen tube to facilitate lung isolation.

Despite various attempts to improve oxygen saturation intraoperatively, a collapsed lung necessitated continuous positive airway pressure mode, resulting in improved oxygen saturation (SpO2) levels. The surgical repair proceeded without complications. However, post procedure, he exhibited an inability to follow commands or perform purposeful movements, prompting the decision to maintain intubation. Subsequently, his double-lumen tube was replaced with a single-lumen tube using a cook catheter under video visualization. Given his esophageal condition, transesophageal echocardiography was not performed. Instead, a transthoracic echocardiogram performed in the intensive care unit (ICU) revealed normal biventricular function, absence of pericardial effusion, and an ejection fraction of approximately 60%. 

**Figure 3:**
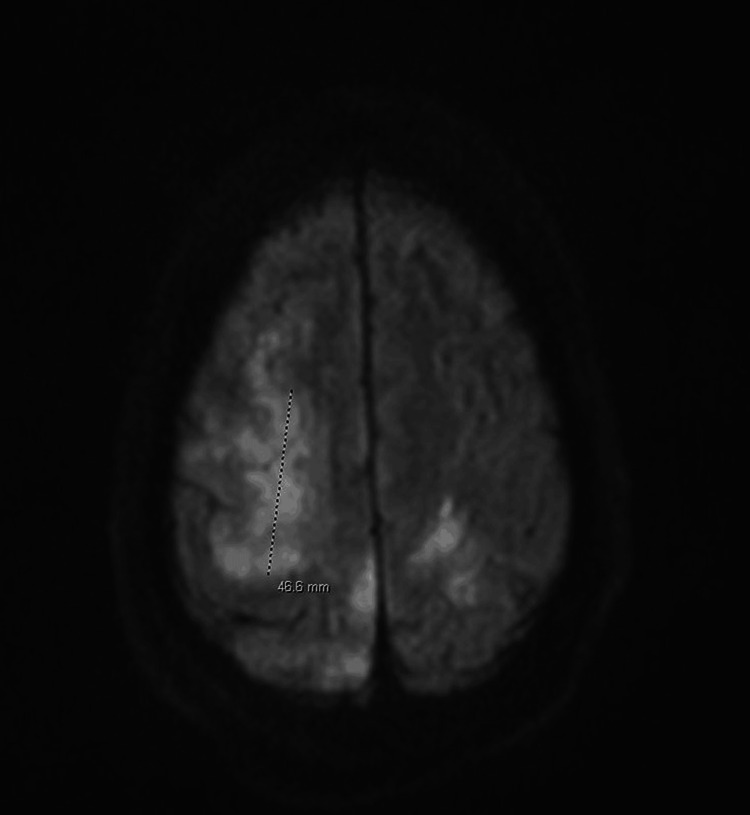
MRI findings suggestive of acute embolic stroke

Within two days, he was successfully extubated in the ICU; however, his left-sided weakness remained. Brain magnetic resonance imaging (MRI) (Figure 3) findings indicated features consistent with an acute embolic stroke affecting multiple areas of the anterior and posterior circulation, along with involvement of the left posterior temporal lobe. Concurrently, blood cultures yielded streptococcal growth, raising concerns for sepsis and potential infective endocarditis. 

His hospitalization was further complicated by the development of an esophageal leak, necessitating the placement of esophageal stents. Following the stabilization of his medical condition, he transitioned to the inpatient rehabilitation unit for comprehensive care. Ultimately, upon achieving medical stability and functional improvement, he was discharged to undergo outpatient physical and occupational therapy. 

## Discussion

Atrial esophageal fistulas are rare complications of catheter ablations, occurring in 0.1% of cases [3]. Although infrequent, they pose a significant risk of mortality, with reported incidences ranging from 40% to 100%. It is imperative for anesthesiologists and surgeons to be cognizant of this complication and to devise a strategic plan for its treatment and the management of any unforeseen events that may arise. 

These fistulas develop when heat generated by the catheter causes thermal damage to the esophagus and compromises its blood supply, resulting in ischemic injury [4]. Esophageal temperature probes are employed to monitor the temperature of the esophagus during the procedure. Studies indicate that temperatures exceeding 41°C are more likely to induce damage [4]. Despite variations in the literature, some studies have suggested a lack of correlation between esophageal injury and esophageal temperature [5]. 

One-lung ventilation is a commonly employed technique in thoracic surgery to optimize visualization of the surgical field. Hypoxia, while infrequent during one-lung ventilation (approximately 4% incidence), may escalate in cases of hemodynamic compromise. It can arise due to right-to-left intrapulmonary shunting and heightened atelectasis in the dependent lung, exacerbating hypoxemia. This decrease in ventilation can stimulate hypoxic pulmonary vasoconstriction, leading to pulmonary vasoconstriction and further exacerbating intrapulmonary shunting. Consequently, this hinders blood flow to the well-oxygenated and dependent lung [6]. 

In the event of hypoxia, the fraction of inspired oxygen (FiO2) should be escalated to 1.0 and the positive end-expiratory pressure (PEEP) should be increased. Verification of the proper positioning of the bronchial blocker or double-lumen tube should be conducted using a flexible fiberoptic bronchoscope. Utilizing passive oxygenation to the non-dependent lung or even application of continuous positive airway pressure, as was employed for our patient during hypoxemia, can facilitate resolution. This mechanism is believed to operate passively, enabling oxygen uptake by the alveoli. If the patient continues to desaturate, then it needs to be discussed with the surgeon, and the patient should be transitioned to two-lung ventilation. 

## Conclusions

While atrial esophageal fistulas constitute a rare yet significant concern in catheter ablation procedures, other potential adverse outcomes encompass vascular complications, stroke or ischemic attack, pericardial effusion, tamponade, pulmonary vein stenosis, and phrenic nerve injury. Physicians and healthcare professionals must remain vigilant regarding such complications. 
